# Widespread convergence towards functional optimization in the lower jaws of crocodile-line archosaurs

**DOI:** 10.1098/rspb.2024.0720

**Published:** 2024-08-21

**Authors:** James R. G. Rawson, William J. Deakin, Thomas L. Stubbs, Thomas J. Smith, Emily J. Rayfield, Phillip C. J. Donoghue

**Affiliations:** ^1^ School of Earth Sciences, University of Bristol, Bristol, UK

**Keywords:** functional landscape, biomechanics, optimality, Crurotarsi, Crocodylomorpha

## Abstract

Extant crocodilian jaws are subject to functional demands induced by feeding and hydrodynamics. However, the morphological and ecological diversity of extinct crocodile-line archosaurs is far greater than that of living crocodilians, featuring repeated convergence towards disparate ecologies including armoured herbivores, terrestrial macropredators and fully marine forms. Crocodile-line archosaurs, therefore, present a fascinating case study for morphological and functional divergence and convergence within a clade across a wide range of ecological scenarios. Here, we build performance landscapes of two-dimensional theoretical jaw shapes to investigate the influence of strength, speed and hydrodynamics in the morphological evolution of crocodile-line archosaur jaws, and test whether ecologically convergent lineages evolved similarly optimal jaw function. Most of the 243 sampled jaw morphologies occupy optimized regions of theoretical morphospace for either rotational efficiency, resistance to Von Mises stress, hydrodynamic efficiency or a trade-off between multiple functions, though some seemingly viable shapes remain unrealized. Jaw speed is optimized only in a narrow region of morphospace whereas many shapes possess optimal jaw strength, which may act as a minimum boundary rather than a strong driver for most taxa. This study highlights the usefulness of theoretical morphology in assessing functional optimality, and for investigating form–function relationships in diverse clades.

## 1. Introduction

**Figure 1.**
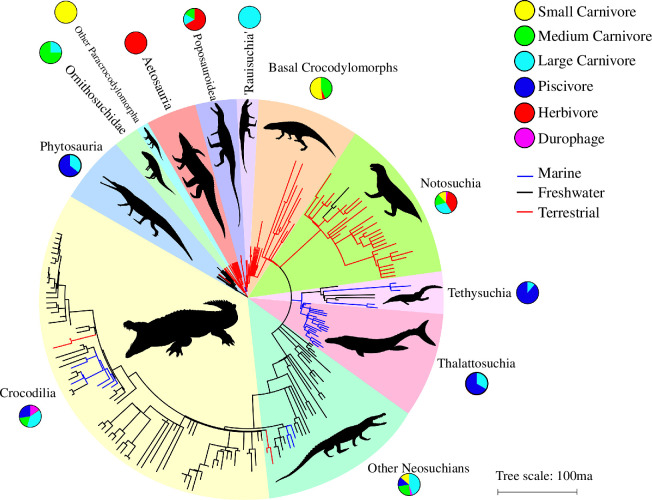
Ecological disparity in crocodile-line archosaurs. Time-calibrated tree showing the sample of crurotarsan taxa assembled in this study. Branch colour denotes habitat groupings: freshwater (black), marine (blue) and terrestrial (red). Pie charts show the distribution of dietary categories within each taxonomic group. The topology used in this tree corresponds to Tree 1 in our study (see electronic supplementary material, table S3, for alternative topologies). Taxon silhouettes are sourced from PhyloPic (see acknowledgements).

Understanding the drivers of morphological change is among the most complex topics in evolutionary biology, and interactions between function, phylogeny and ecology are often difficult to disentangle [1]. Modern representatives of the clade Crurotarsi, the crocodile-line archosaurs, have been a staple of form–function studies for decades, but extinct crurotarsan lineages occupied a much greater range of ecological niches than their extant relatives [2,3]. Living crocodilians spend most of their time in freshwater [4] but many crurotarsans have at some point occupied terrestrial and marine habitats (figure 1). Terrestrial forms include many non-crocodylomorph crurotarsans and basal crocodylomorphs [5], as well as notosuchians that radiated considerably in Cretaceous Gondwana [6]. Neosuchians also returned to a land-living lifestyle on two occasions, within Planocraniidae and Mekosuchinae [5]. These terrestrial forms show considerable disparity in diet (figure 1) with Triassic aetosaurs and poposauroids showing adaptations for herbivory and omnivory [7,8], traits which also evolved independently in numerous notosuchians [9]. Other terrestrial crurotarsans occupied top predatory niches, from Triassic ‘rauisuchians’ [10] to huge sebecid notosuchians in the Cenozoic [11,12]. Crurotarsans have shifted between freshwater and marine habitats on at least nine occasions, with notable marine radiations within Gavialoidea and multiple times within Tethysuchia [5]. The most drastic shift to marine life is seen in thalattosuchians; metriorhynchoids, in particular, possessed paddle-like limbs, lacked the heavy osteoderms seen in other crocodylomorphs and were most likely viviparous [13]. Longirostrine jaws that were likely specialized to catch small prey have evolved convergently in at least eleven lineages of aquatic crocodyliforms [14], both freshwater and marine. Other unusual forms have evolved multiple times within the clade, such as the ‘duck-faced’ stomatosuchids (Cretaceous neosuchians) and *Mourasuchus* (a Miocene alligatoroid) that possessed long, broad snouts possibly adapted for gulping large quantities of small prey [15]. Crurotarsans, therefore, provide a wealth of examples to test the effects of morphological and functional divergence and convergence across very disparate ecological settings.

Previous studies of morphological and functional evolution across crurotarsan history have used the lower jaw as a proxy of ecomorphological and biomechanical disparity [2,16]. Jaws are an excellent model for testing form–function hypotheses: they are almost ubiquitous among vertebrates and are specialized for few functions other than feeding, giving them a higher evolutionary plasticity than skulls [17]. Jaw morphology in modern crocodilians is likely the result of a trade-off between hydrodynamics, jaw strength and jaw closure speed [18,19], but it is unclear whether this applies to extinct species with vastly different ecologies. Environment likely plays an important role in determining crurotarsan morphology. Fully aquatic taxa show high levels of convergence due to increased hydrodynamic constraint [20], while terrestrial lineages are free to expand their morphological diversity [16]. A correlation between morphology and diet is supported by the categorization of snout shape among extant [21] and extinct [14] species, but relying on gross morphology alone may obscure functional disparity between jaws [22,23]. Biomechanical evidence supports these hypotheses of form–function relationships [20,22,23], but in many lineages this evidence stems from a single or small number of taxa and many clades lack any quantitative testing. This is especially true of basal crurotarsans outside Crocodylomorpha (figure 1), which have undergone little functional and morphological study. Their distribution among the proposed cranial morphotypes remains largely unknown and quantitative biomechanical analyses on these lineages have so far been confined to aetosaurs [24,25], phytosaurs [26], ornithosuchids [27] and the ‘ostrich-like’ shuvosaurid *Effigia okeeffeae* [7].

Here, we quantitatively investigate the effect of morphological change and varying ecology on the functional optimization of crurotarsan jaws across their long evolutionary history. We employ a theoretical morphology approach to characterize the function of two-dimensional realized and unrealized lateral jaw shapes. We use these shapes as an abstraction of empirical crurotarsan jaws, exploring whether crocodile-line archosaurs that show morphological convergence also reach shared functional solutions in similar ecological scenarios, and whether these solutions are functionally optimal for fast closure speed, stress resistance, hydrodynamic efficiency or a trade-off between multiple functions. Following [28], we used an empirical sample of two-dimensional lateral jaw outlines from 243 crurotarsan species to create a theoretical morphospace, analysing the functional performance of individual theoretical shapes to create a ‘performance surface’ that shows how changes to these abstractions of jaw morphology affect functional output. Theoretical morphospaces, unlike empirical ones, allow comparison of occupied and unoccupied morphospaces to test whether taxa preferentially evolve shapes that mirror theoretical shapes with the most optimal performance. They also quantify the functional performance regions of shape space that are poorly sampled for empirical taxa, a pervasive problem with the fossil record [29]. We hypothesize that ecologically convergent crurotarsans (figure 1) will also show convergent functional optimization, and that the morphology they converge towards will be more optimized for ecologically relevant functions than unrealized shapes. For example, we expect taxa that hunt small prey in aquatic, and especially marine, environments to convergently evolve jaws more similar to theoretical shapes that are optimized for a trade-off between closure speed and hydrodynamics than to shapes in any other region of theoretical morphospace, realized or not. We also expect the lower jaws of taxa that have independently evolved herbivory to be more similar to shapes optimized for increased stress resistance. However, functional performance is not equivalent to fitness, and we may find that some jaw shapes are not optimal, and that some optimal shapes may never have evolved.

## 2. Material and methods

### (a) Jaw shape and taxon data

We gathered lateral jaw images from 243 species and dorsal data from 160 species (electronic supplementary material, table S1; dorsal morphospace shown in electronic supplementary material, figure S10). In addition to shape data, ecological data pertaining to both habitat and diet were obtained for each species. Habitat data were gathered from the literature, including from previous large-scale studies on crurotarsan habitat occupation [2,5,30] (electronic supplementary material, table S1). Where habitat data were unavailable, habitat was inferred from postcranial morphology, depositional environment and certain features of cranial morphology that are unlikely to covary with lower jaw morphology (e.g. position of orbits/nares). Taxa were categorized as terrestrial, freshwater or marine based on predominant environment, meaning that taxa such as *Crocodylus porosus* that occasionally make forays into coastal marine habitats are coded here as freshwater. No distinction was made between fully aquatic marine forms such as metriorhynchoids and semi-aquatic marine forms such as teleosauroids, given that current evidence suggests that these two clades, and likely other marine lineages, occupied overlapping habitats within the marine realm [31]. Dietary data were collated using factors independent of jaw morphology such as preserved stomach contents and tooth morphology. Inferring dietary data in fossil taxa naturally presents difficulties, but such categories have been inferred in previous large-scale crocodylomorph studies [2,3]. Taxa were assigned either piscivore, facultative herbivore (including omnivores), durophage or carnivore, with carnivore being further split into small/insectivore (<10 cm mandible length), medium (10−30 cm mandible length) and large (>30 cm mandible length). A breakdown of sample size for each taxonomic and ecological group can be found in electronic supplementary material, table S2.

### (b) Generating the theoretical morphospace

The pipeline we employed [28] uses elliptical Fourier analysis (EFA) to construct a theoretical morphospace of jaw shapes [32]. EFA uses landmarks to characterize the shape of a closed two-dimensional loop using a series of elliptic harmonics [33], each of which has less impact on shape determination than its predecessor. The mathematical formulae of these harmonics, standardized for size and orientation with the first harmonic along the *x*-axis, allow shape variation to be measured quantitatively. The EFA and all subsequent functional analyses were performed in MATLAB [34], the underlying code and functions for which are available from GitHub (https://github.com/w-j-deakin/theofun). The MATLAB script used for this study, alongside all the tif files of jaw outlines in our sample, can be found here: https://doi.org/10.5061/dryad.dbrv15f6n [35]. Lateral jaw images were prepared for EFA in Inkscape [36]. Teeth were omitted due to their disproportionate influence on shape variation and poor preservation in many taxa. Images were then imported into ImageJ [37], transformed into single-pixel binary outlines and saved as tif files to be inputted into MATLAB. To ensure that the morphological variation in the jaw outlines was captured sufficiently, a sensitivity analysis was performed across our EFA data to find the optimum number of landmarks (600) for each outline (electronic supplementary material, figure S1). A principal component analysis (PCA) was then performed on the dataset of continuous characters generated by EFA to build an empirical morphospace, from which harmonic data were extracted at regular intervals. This was used to plot a grid of 500 theoretical jaw shapes that varied along the PC axes to include all realized jaw forms plus an additional amount equivalent to 20% of the range of PC1. An identical approach was applied to the dorsal jaw data. We also tested the sensitivity of the theoretical shape space to the removal of taxa from our empirical sample (electronic supplementary material, figure S2).

### (c) Functional analyses

Resistance against loading during feeding has been proposed to be an important adaptive feature in lower jaws [17,38], and here we use Von Mises stresses (VMS) calculated using finite element analysis (FEA) as a proxy for jaw strength. FEA was carried out on each theoretical shape using the method described in Deakin *et al*. [28], where meshes of 2500 triangles were analysed using a simple two-dimensional constant strain triangle FEA algorithm in MATLAB. Theoretical jaws were modelled as a uniformly thick plate scaled to an equal surface area with a Young’s modulus of 2 × 10^9^ Pa and a Poisson’s ratio of 0.3 [39]. Theoretical morphologies lack pre-defined jaw joints, muscle forces and homologous bite positions, so these locations and input forces must be designated within the functional analyses using shape properties. Here, the left- and rightmost nodes with near vertical normals were marked as the jaw joint and the end of the tooth row, respectively. The muscle input force was initially positioned at one-third of the length of the jaw from the joint. To account for unknown variation in muscle force vectors, we bootstrapped our lower jaw models over 1000 pseudo-randomizations of force node position (up to 5% of total outline length on either side of the initial node placement) and force direction (45° on either side of the force node normal) per FE model to generate 95% confidence intervals, producing 500 000 models for each performance surface. In all cases, the force was aligned directly on the midsagittal plane to avoid any mediolateral force component acting on our two-dimensional models. Performance surfaces were plotted using median VMS to avoid outliers measured at constraints [28]. Comparisons with previously published FE data using three-dimensional models of extant crocodilians were performed to assess the validity of our two-dimensional abstracted models, which can be found in the electronic supplementary material, figure S3.

Rotational efficiency is defined as the velocity of the jaw tip when rotating about an axis given one unit of energy, which in this case acts as a proxy for jaw closure speed. Closure speed has long been considered an adaptive feature in lower jaws, often associated with catching small or fast-moving prey [14,20]. In our analysis, rotational efficiency was calculated for each shape in the theoretical morphospace by finding the velocity of the bite point when given a rotational energy of one joule (*v*) using the following equation:


$$v=L\sqrt{\frac{2}{I}},$$

where *L* is the length of the bite point away from the rotational axis and *I* is the moment of inertia at the bite point [28]. Moment of inertia, a value that denotes the torque required for a specific rotational acceleration, was calculated using the element mass (*m*), distance from the rotational axis to the element centre of mass (*r*) and the number of elements (*N*) using the following equation:


$$I\;\approx \;\sum_{i=1}^{N}m_{i}r_{i}^{2}.$$

Models for rotational efficiency were also bootstrapped over 1000 randomizations that varied the location of the jaw joint and jaw tip (5% of total outline length on either side of the initial node placement) to calculate 95% confidence intervals from which a median performance surface could be plotted.

Modern crocodilians use lateral head movements to capture prey [18,40,41], and evidence suggests many extinct crurotarsans fed in the same manner [23,42]. Drag scales linearly with the area exposed in orthographic (in this case lateral) view, so relative jaw area provides a proxy for estimating relative levels of drag as shapes move through the fluid. Jaws with a higher lateral area will exhibit higher form drag during lateral head sweeps. Estimating drag from simple two-dimensional outlines will inherently fail to account for shape variation in all planes, but mediolateral shape variation is known to be relatively limited in the jaws of crocodile-like archosaurs [33,43] and should be highlighted by our dorsal morphospace (electronic supplementary material, S6). Relative jaw area, essentially a measure of aspect ratio calculated when all outlines were scaled to the first harmonic (i.e. length), was calculated in MATLAB [28] using the polyarea function.

### (d) Pareto optimality

Adaptive landscapes are commonly constructed using maximum likelihood or regression, based on inputted or empirically derived measures of fitness [44,45]. However, this relies on the assumption that a single fitness metric applies to all the studied shapes. These methods also have limits when using functional metrics for which performance across shape space is hard to predict and may not be well characterized by a simple polynomial. In this study, we performed Pareto optimality analyses on our three generated performance surfaces using the methods first described in [28] to produce three two-dimensional surfaces for each pair of tested functions and a single surface combining all three functional metrics. This method ranks the optimality of each theoretical jaw shape using a modified Golberg Pareto ranking system to recover a subset of theoretical shapes that are Pareto optimal (the definition being when no theoretical shapes perform better than or equal to this subset for the tested functional metrics). This Pareto optimal subset is removed from the sample and the calculation can thus be repeated iteratively, generating a ranking of theoretical shapes from zero (suboptimal) to one (where one is Pareto optimal). An optimality surface plotting these Pareto rankings was used to highlight areas of the theoretical lower jaw morphospace which maximize multiple functions simultaneously and are, therefore, well adapted for functional trade-offs.

### (e) Statistical testing of morphological convergence

To test whether crurotarsans of different environments, diets and taxonomic groups occupy distinct regions of morphospace, a phylogenetic MANOVA was carried out using the procD.pgls function from the geomorph package [46] in R [47] over 10 000 iterations. We used our harmonic data of empirical taxa as the dependent variable and one of our ecological/taxonomic groups as the independent variable in each analysis. Eight informal supertrees of crurotarsan relationships were constructed to perform this analysis to test the effects of different phylogenetic hypotheses (see electronic supplementary material). We then tested for significant differences between each of our categories given the impact of phylogeny using the pairwise function in the RRPP package [48]. Rejection of the null hypothesis (*p *< 0.05) indicated that jaw morphology differed significantly between the tested groups. Additionally, PC coordinates of each empirical lateral jaw outline and ancestral nodes obtained using the gm.prcomp function from geomorph [46] were used to calculate a *Ct*1 statistic for each pairwise comparison of taxa to quantitatively assess morphological convergence in our morphospace. *Ct*1 is an improved and updated version of the *C*1 statistic used for measuring phenotypic convergence [49] that minimizes the possibility of misidentifying divergent taxa as convergent [50]. We also categorized these pairwise statistical comparisons using our ecological variables (environment and diet) to compare morphological convergence in crurotarsans with different ecologies.

## 3. Results

### (a) Theoretical morphospace

**Figure 2.**
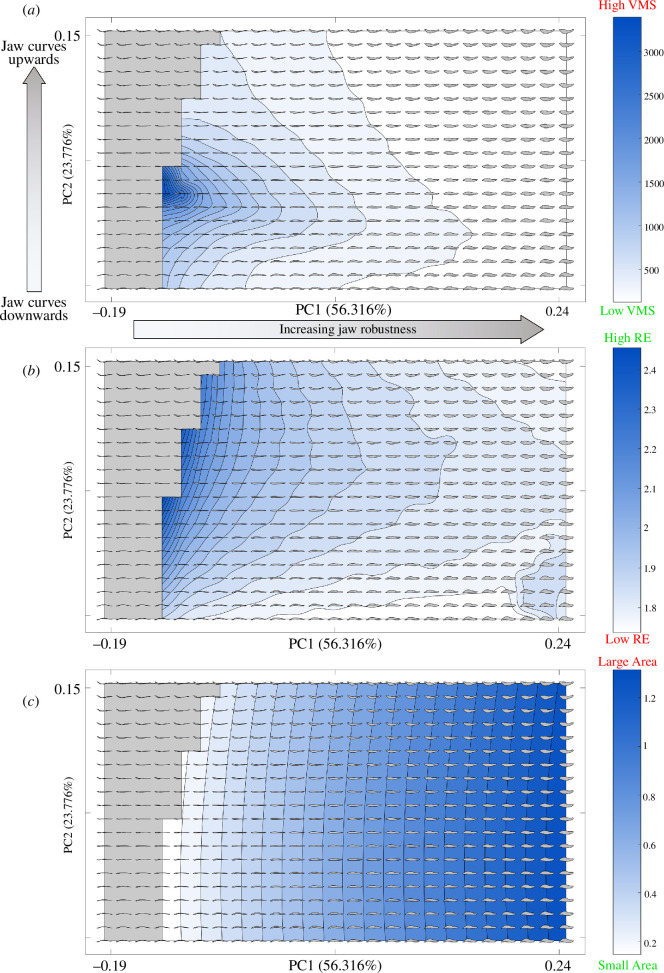
Performance surfaces of functions tested: (*a*) Von Mises stress, (*b*) rotational efficiency and (*c*) jaw area, for (*a,b*) showing the mean values from 1000 iterations with pseudo-randomly sampled input parameters within a given range (see §2). Areas with darker blue denote theoretical lateral lower jaw outlines with higher values for tested functions that are interpreted as showing reduced (*a,c*) or increased (*b*) fitness. Theoretical shapes across the morphospace are superimposed with the anterior end facing right. Jaw silhouettes are scaled to the first harmonic (i.e. length) in the figure; note that this does not reflect their relative size within the functional analyses. Grey areas denote impossible morphospace.

The theoretical morphospace highlights the main axes of variation within our sample of generated two-dimensional theoretical jaw shapes. PC1 (56.32% of variance) was characterized by a change in relative robustness of the tooth-bearing region compared with the posterior region and overall jaw depth, shifting from gracile jaws towards more robust jaws as the value for PC1 increased (figure 2). PC2 (23.78% of variance) was associated with the curvature of the posterior section of the jaw and the position of the jaw joint relative to the tooth row. Theoretical jaws with negative PC2 values showed dorsally convex profiles with ventrally placed jaw joints, while jaws with positive PC2 showed ventrally convex profiles and dorsally placed jaw joints. These first two principal axes, therefore, capture 80% of the total lateral variation in crurotarsan jaw shapes and were used in all subsequent analyses. PC3 (5.00% of variance) was mainly characterized by the distribution of mass around the jaw joint and the curvature of the jaw tip. The shapes along the principal axes in our theoretical morphospace varied similarly to previous empirical studies using two-dimensional lateral outlines of crurotarsan lower jaws [16]. Some shapes at the negative end of PC1 were recovered within impossible morphospace: the closed loops were self-intersecting and thus impossible to form in reality.

### (b) Functional analyses

Testing the mechanical performance of each individual two-dimensional theoretical shape allowed us to explore functional changes across the theoretical morphospace. Resistance to VMS varies across PC1 and PC2 (figure 2*a*), with the highest stresses found at lower PC1 values/intermediate PC2 values, close to impossible morphospace. Gracile jaws are, therefore, less able to dissipate VMS across their surface than more robust jaws [20,22]. Theoretical shapes at the extremes of PC2 showed lower levels of stress than those of intermediate PC2 values, suggesting that increased jaw curvature is an important factor in dissipating stresses, regardless of direction. Stress resistance increases rapidly away from the worst performing shapes so that a large proportion of jaw shapes show a similarly high ability to dissipate VMS. Rotational efficiency, our proxy for jaw closure speed, mainly varies across PC1 (figure 2*b*): high rotational efficiency is found closer to negative values and peaks on the boundaries of impossible morphospace. This indicates that gracile jaw shapes are better optimized for fast jaw closure than robust jaws, in accordance with previous morphological and biomechanical hypotheses [20,32]. Our results suggest a clear trade-off between jaw strength and jaw speed along PC1 as jaws shift from being more gracile (high VMS, high RE) to more robust (low VMS, low RE). Jaw area, which is used here in the two-dimensional lateral profile as a proxy for hydrodynamic efficiency during lateral sweeps, was minimized towards negative PC1 (figure 2*c*), meaning that jaws which are more gracile and slightly upwardly curved are more efficient at sweeping laterally when feeding in water.

**Figure 3.**
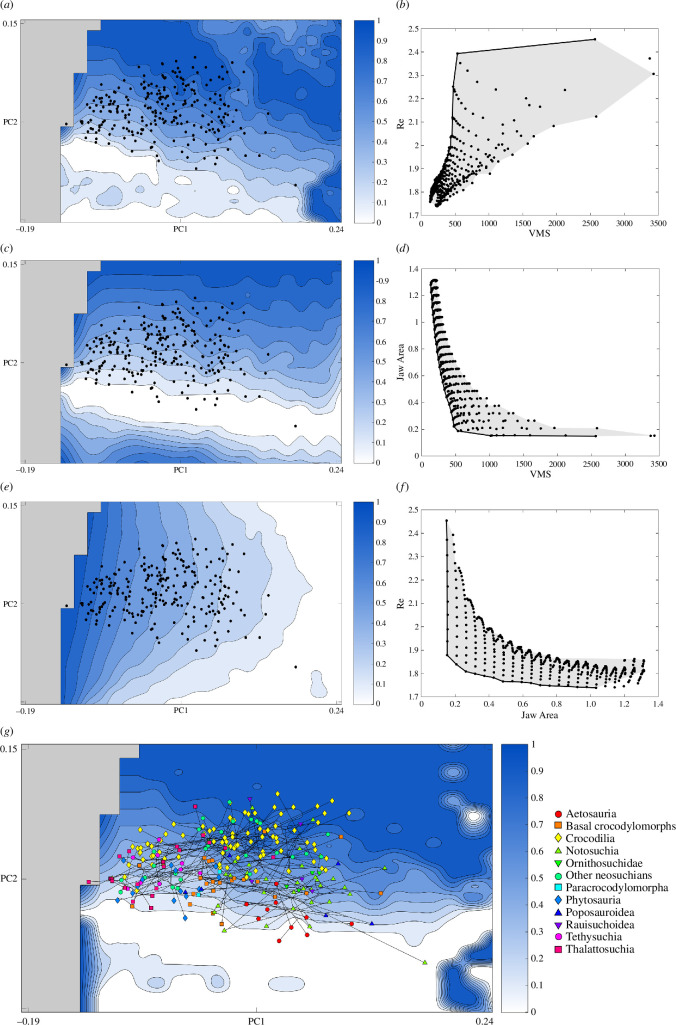
Pareto optimality. (*a,c,e*) Combined performance surfaces for each pair of tested functional metrics: VMS and rotational efficiency (*a*), VMS and jaw area (*c*) and rotational efficiency and jaw area (*e*). Areas of morphospace with higher Pareto ranks are shown by darker colours. Grey areas indicate impossible morphospace and black dots (*n* = 243) denote the positions of empirical taxa. (*b,d,f*) Performance space plotted using the functional output of each theoretical jaw shape, corresponding to the performance surface to each plot’s immediate left. Each theoretical two-dimensional lower jaw shape is represented by a black dot, showing the heterogeneity of functional occupation. The Pareto front is shown as a solid black line and the grey area represents the range of possible solutions. (*g*) Performance surface combining all three functional metrics overlayed with a phylomorphospace, with empirical jaws split by taxonomic group. Topology corresponds to Tree 1 (electronic supplementary material, table S3, and see figures S5 and S6 for alternative topologies).

Testing the Pareto optimality of pairs of performance surfaces shows that theoretical jaw shapes which are optimized for a trade-off between rotational efficiency and VMS resistance lie in the high PC2 regions, though shapes in the high PC1/low PC2 region also perform very well (figure 3*a*). A high proportion of theoretical shapes cluster close to the Pareto front (the set of points in morphospace that rank as Pareto optimal) in areas of low stress and low to intermediate rotational efficiency (figure 3*b*). A notable feature of the Pareto front in this trade-off is that achieving the highest levels of rotational efficiency incurs a steep cost in jaw strength, and very few theoretical shapes occupy this region. The most optimal two-dimensional lateral shapes for a trade-off between jaw strength and hydrodynamic efficiency lie on the extremes of PC2 due to the penalty of a straight lateral profile on stress dissipation (figure 3*c*). When projected onto the theoretical shapes, most realized jaws occupy areas of this landscape with poor to intermediate optimality, with most optimal regions remaining unoccupied. A trade-off between rotational efficiency and hydrodynamic efficiency (figure 3*e*) is optimized at low PC1 values and intermediate to high PC2 values, which correspond to the two-dimensional outlines of more gracile jaws that show no or slightly upward curvature. Most of the theoretical shapes sit close to the Pareto front in areas of low rotational efficiency, with relatively few showing high rotational efficiency (figure 3*f*). In the landscape that combines all three surfaces, highlighting areas of Pareto optimality for all three functional traits (figure 3*g*), the bulk of the Pareto optimal area sits towards the positive end of PC2. Within the generally poorly optimized negative PC2 space, the extremes of PC1 also show high optimality. Apart from these isolated areas, jaws that curve downwards in lateral view are poorly optimized for a trade-off between all our tested functions.

### (c) Distribution of taxa in theoretical space

**Figure 4.**
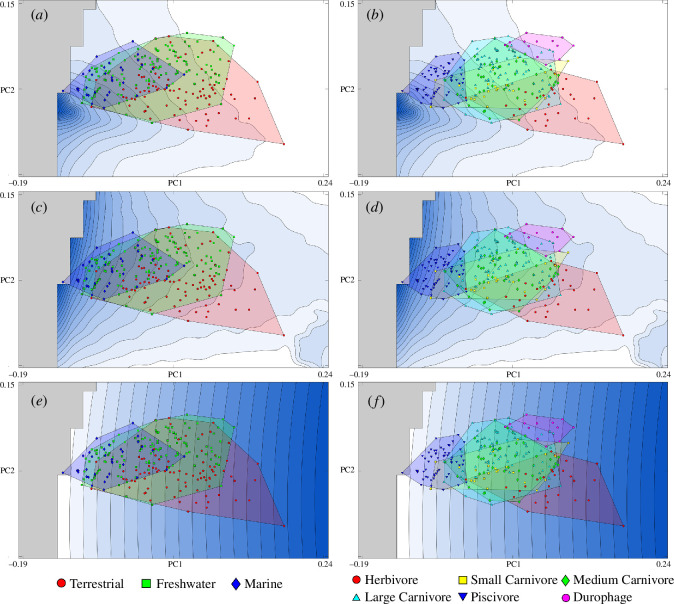
Effects of ecology on functional optimization of two-dimensional lower jaw shapes. Performance surfaces of functions tested (as in figure 2): Von Mises stress (*a,b*), rotational efficiency (*c,d*) and jaw area (*e,f*), overlayed with empirical taxa grouped by habitat (*a,c,e*) and diet (*b,d,f*). Areas of morphospace with higher values for tested functions are shown by darker colours as in figure 2. Grey areas denote impossible morphospace. Equivalent plots for combined performance surfaces, as in figure 3, are provided in electronic supplementary material, figure S9.

Taxa occupying different habitats (figure 4*a,c,e*) show overlap in the morphospace, and the groups are often statistically indistinguishable when accounting for phylogeny (electronic supplementary material, table S4). Moreover, our phylogenetic MANOVA did not recover a significant relationship between habitat and jaw shape for our tested trees. Marine taxa take up the smallest area of morphospace and lie towards the negative end of PC1, with *Pelagosaurus* plotting within impossible space. This is not an error but an artefact of dimension reduction, where taxa may appear to lie in impossible space when viewing the data in the PC1/PC2 plane. The lateral jaw outlines of marine taxa closely match theoretical shapes that perform well for rotational efficiency and hydrodynamics (figure 4*c,* *e*), as well as a trade-off between the two (figure 3*e*). Marine taxa that strongly resemble shapes with higher PC1 values, and somewhat greater jaw strength (figure 4*a*), mostly consist of large metriorhynchoids such as *Dakosaurus* and *Suchodus* but also the robust tomistomine *Maroccosuchus*. Overall convergence between marine taxa is not particularly elevated when measured using the *Ct*1 metric (electronic supplementary material, figure S8*a*), though select taxa do show high convergence with each other as well as with some freshwater taxa. Terrestrial taxa are by far the most morphologically disparate habitat grouping; their two-dimensional morphospace occupation overlaps all but the most gracile aquatic taxa and extends into the high PC1/low PC2 region. Taxa in this area of morphospace mostly consist of aetosaurs and notosuchians such as *Simosuchus* and *Malawisuchus* but also include the protosuchid Kayenta form (UCMP 125871) and the poposauroid *Lotosaurus*. Terrestrial taxa show no clear trend of morphological convergence (electronic supplementary material, figure S8*a*). Freshwater taxa fall mostly in the centre of occupied morphospace, with many representatives overlapping almost entirely with marine and terrestrial groups.

A similar degree of overlap emerges when projections of empirical taxa onto the morphospace are split by proposed diet (figure 4*b,d,f*). We found that diet had a significant effect on the lateral jaw shape of empirical taxa when correcting for phylogeny for all but one of our tested trees (Tree 5; see electronic supplementary material, table S3). Some herbivorous taxa occupy their own distinct region of morphospace, matching theoretical outlines of jaws that are more robust, dorsally convex and possess a jaw joint roughly in line with the tooth row. One notable exception is *Shuvosaurus*, the jaw of which is far more gracile than any other herbivore included here. Piscivorous taxa are fairly restricted in two-dimensional lateral morphospace and show the greatest degree of morphological convergence of any dietary category (electronic supplementary material, figure S8*b*); all jaws in this category are highly gracile and show little dorsoventral curvature. Carnivorous taxa of all sizes overlap around the central region of the occupied morphospace, though whether this overlap was significant depended on the tree being used in the phylogenetic MANOVA (electronic supplementary material, table S4). Large carnivores also appear to show some morphological convergence with each other and with medium carnivores (electronic supplementary material, figure S8*b*). Durophagous taxa, represented by several genera of shell-feeding alligatoroids, the basal eusuchian *Iharkutosuchus* and the terrestrial *Mekosuchus inexpectatus*, show a convergent exploration into shapes which are ventrally convex, have dorsally placed jaw joints and are moderately robust, though not as robust as some herbivores. A few carnivores exhibit gracile jaws that overlap strongly with piscivorous taxa, those being the terrestrial insectivorous sphenosuchians *Terrestrisuchus* and *Litargosuchus*, the macropredatory aquatic teleosaurid *Machimosaurus* and the paralligatorid *Rugosuchus*.

## 4. Discussion

### (a) Occupation of the theoretical morphospace

Using the theoretical morphospace as an abstraction of real-world variation in crurotarsan jaw shape allows us to assess why some regions of morphospace are occupied over others. Some of this absence can be explained by impossible morphologies (jaw outlines at the negative end of PC1 show self-intersection; figure 2), but we must ask why other seemingly viable shapes were never realized by empirical taxa. Significant phylogenetic signal in our sample of empirical shapes for all tested tree topologies (electronic supplementary material, table S3) indicates that phylogeny has been a driver of morphological evolution in crurotarsan jaws [1,51], though this signal is fairly weak across all of the tested trees.

The performance of our two-dimensional theoretical shapes can inform us of functional differences between realized and unrealized jaws, and our results suggest that function is also a driver of morphology. Very few taxa match the lateral outlines of theoretical shapes that lie in the least optimal morphospace, at least in our standardized sampling window. The restriction shown by empirical taxa against morphospace with significant functional penalties indicates that jaw speed, strength and hydrodynamics do produce an adaptive signal in the jaw morphology of crurotarsans, at least in these two-dimensional abstractions. The steep reduction in rotational efficiency moving away from impossible morphospace and relatively poor performance for most theoretical shapes (figure 2*b*) means that jaw speed has the potential to greatly restrict the range of functionally optimal shapes, assuming strong positive selection. By contrast, stress resistance is optimal or near optimal in a large range of theoretical two-dimensional shapes (figure 2*a*). A trade-off between stress resistance and rotational efficiency favours jaws with an upward curvature (figure 3*a*), since downward curving jaws incur a minor speed cost and straight jaws incur a more major cost to strength. While taxa do not appear to be strongly constrained to the areas of morphospace that optimize this trade-off, they do entirely avoid the most poorly optimized areas. Large areas of morphospace where functional optimality is invariable, as is seen for rotational efficiency and jaw strength, have implications for estimations of mechanical disparity [52], a topic that has been given some attention in Crurotarsi. Previous studies using two-dimensional representations of crurotarsan morphology have compared gross shape or simple lever ratios to estimate variation in function [2,14], but our results from testing theoretical shapes imply that these measures may not be fully representative of true mechanical disparity.

An interesting pattern emerges when considering how the distribution of empirical taxa compares to our theoretical landscape of jaw strength. Though realized morphologies do avoid the areas of highest stress (figure 2*a*), they do not otherwise closely follow the other contours of the landscape. It seems unusual that jaw strength, often hypothesized to be a major adaptive feature of crurotarsans [21,22], does not appear to greatly constrain their lateral jaw morphology. It may be that selection for jaw strength manifests largely as a constraint rather than as a driver, where all but the weakest jaws can withstand loading during feeding and so all have similar adaptive advantages. There would, therefore, be little need to sacrifice performance in speed or hydrodynamics to make gains in jaw strength, especially for taxa where those functions are important. We should also consider the effect that size can have on functional performance. Size correction is a vital step in the theoretical morphology approach we employ here, but body size variation in realized crurotarsan jaws will affect functional output. Larger bodied taxa can generate higher absolute bite forces and distribute equivalent stresses across a larger surface area than small-bodied taxa, and so may face less constraint on jaw shape when feeding on similar diets. A few realized jaws do correspond to theoretical shapes showing increased strength at the cost of other functions though, which may be a feature of ecology (see §4b). Many realized jaw outlines lie in the optimal region for a trade-off between jaw speed and area (figure 3*e*), which is expected for aquatic and semi-aquatic taxa where hydrodynamic performance is important [18].

Although the distribution of realized jaw outlines largely corresponds to the functional optimality of their theoretical abstractions, some regions of unoccupied morphospace do not appear to bear a significant adaptive cost. Morphospace at low PC1/high PC2 and high PC1/low PC2 shows good performance for strength and speed (figure 3*a*), yet these theoretical models do not resemble the outlines of any of our studied taxa. There is a cost to hydrodynamic efficiency at high PC1/low PC2 (figure 2*c*), but this should not be an issue for terrestrial taxa. It may be that these shapes are difficult to evolve due to constraints on skull shape. Although the jaw is treated here as an independent unit, we must consider that the jaws and cranium often function as a single system. Confounding factors from sensory organs or muscle attachments may have a knock-on effect on jaw morphology, though these effects will have far less effect on the jaw than on the cranium itself [17]. It may also be that there is no adaptive reason why these shapes did not manifest in nature and that there may simply have been insufficient time for them to evolve in this lineage.

### (b) Functional convergence among Crurotarsi

Our results suggest that functional convergence is common throughout crurotarsan evolution (electronic supplementary material, S8 and table S3). The tight restriction on theoretical jaw shapes that perform well for rotational efficiency may result in functional constraints for aquatic taxa that face pressure to maximize both hydrodynamic performance and speed. Gracile two-dimensional outlines with little curvature produce high values for both metrics while also avoiding the weakest jaw shapes (figure 2*a*), though the smaller area for muscle attachment and resultant weaker bite force in the real jaws of longirostrine taxa [20] mean they are less likely to experience high stresses in any case. Longirostrine forms are convergently reached by multiple clades (electronic supplementary material, figure S4), all of which show adaptations for aquatic (or semi-aquatic) lifestyles and piscivory. Indeed, the high levels of measured morphological convergence between piscivorous taxa compared with other dietary (and environmental) categories (electronic supplementary material, figure S8) indicate that functional constraint has had a particularly notable influence on the morphological evolution of fish-eating crurotarsans.

Though jaw strength does not appear to be a strong constraint overall, certain ecologies may show a demand for increased stress resistance. The lateral outlines of durophagous species are most comparable to theoretical shapes that show some of the highest resistance to VMS of any shape within the morphospace (figure 4*b*), and elevated levels of convergence from multiple lineages (electronic supplementary material, S4 and S8*b*) indicate that this is a functional adaptation. This difference is not particularly strong though, with durophages overlapping other dietary categories and not being significantly different when phylogeny was accounted for (electronic supplementary material, table S4). Durophagous taxa feeding on sessile food such as molluscs have no need to evolve fast-closing jaws, and so their exploration into stronger functional morphospace may be due to a relaxation of selection for jaw speed, with a lesser positive pressure from jaw strength. Adaptations for producing high bite forces also secondarily affect stress resistance; a more dorsally placed cranio-mandibular joint increases both muscle moment arms [53] and jaw curvature, and a larger angular with more muscle attachment [54] increases overall robustness. This may result in the jaws of durophagous taxa being adapted to resist very high stresses, despite strength not being the primary driver behind jaw morphology. This is likely the case in extant lizards, where populations feeding on a higher proportion of hard material show more robust mandibles with larger muscle attachment sites for bite force generation [55]. Notably, lizards that feed on shelled invertebrates increase the size of different masticatory muscles compared with those which feed on more plant material, which is likely an adaptation for generating high bite force at wide and low gapes, respectively. A similar pattern in crurotarsans may explain why durophagous taxa do not overlap with herbivorous taxa in the morphospace (figure 4), despite both feeding on mechanically resistant material.

Herbivorous taxa in our sample are associated with theoretical shapes with lower rotational efficiency (figure 4*d*) and higher jaw strength (figure 4*b*), though the increase in the latter is minor. Herbivory is associated with relatively shorter mandibles, greater mechanical advantage and reduced joint reaction forces in lizards [56–58], which is hypothesized to relate to a need for higher bite forces. At least some herbivorous crurotarsans engaged in more complex oral food processing than extant herbivorous reptiles [53,59] but their tooth morphology and wear patterns indicate they are at least somewhat analogous [53]. The presence of terrestrial carnivores in areas of morphospace where shapes do not maximize strength and instead maintain higher values of rotational efficiency suggests that jaw strength is not an overwhelming selective pressure in terrestrial environments and that the higher strength observed in herbivores is a dietary signal. A correlation between herbivory and morphology does not necessarily imply a strong adaptive signal though, and relaxation of selection for fast jaws, vital for prey capture in carnivores, may also be responsible. The toothless jaws and small head of *Shuvosaurus*, which possesses a more gracile jaw than other herbivores, may suggest a more selective mode of feeding [60].

Hydrodynamic efficiency likely imposes some functional constraints. Macropredators all possess stronger jaws than their generalist or piscivorous relatives but marine forms like *Dakosaurus* and *Suchodus* never reach the same level of robustness as terrestrial macropredators like the larger rauisuchians and baurusuchids (figure 4; electronic supplementary material, S3) despite showing morphological convergence with these taxa (electronic supplementary material, figure S7). Freshwater macropredators do not seem to be constrained in the same way as marine taxa, which could suggest a lower demand for hydrodynamic performance in species that ambush terrestrial prey. Alternatively, this pattern could be explained by differences in skull shape. The terrestrial and marine macropredators in our sample possess domed, oreinirostral skulls, which are well suited to dissipate stress and produce higher bite forces [61,62]. In contrast, almost all the freshwater macropredators in our dataset are crocodilians, which possess more platyrostral skulls [63] better suited to hydrodynamic efficiency during lateral head sweeps when feeding [18]. Crocodilians have been able to circumvent much of the cost to strength normally associated with a platyrostral skull by evolving a secondary palate to better resist torsional stress [61], allowing them to evolve skull shapes adapted for both hydrodynamics and strength. Given that the heads of crocodilians are already well adapted to moving through the water due to their platyrostral skulls, they may experience less pressure to optimize hydrodynamic efficiency in their lower jaws compared with oreinirostral marine macropredators. Interestingly, crocodilians and other neosuchians generally sit closer to theoretical shapes optimized for a trade-off between all three tested functions than other crurotarsans (figure 3*g*), except for a cluster of longirostrine forms more similar to shapes suited to hydrodynamics and speed. Neosuchians, therefore, may show a shift towards jaw shapes more optimized for a trade-off between speed, strength and hydrodynamics compared with those of other crurotarsans, with multiple forays into longirostrine morphospace in more piscivorous taxa.

Overall, we observed lower levels of morphological convergence within environmental groupings than within dietary groupings, and especially within piscivorous species (electronic supplementary material, figure S8). This suggests that, despite the influence of hydrodynamic constraint in restricting the range of optimal morphologies for aquatic taxa, dietary signal is the stronger indicator of morphological evolution in the lower jaw of crurotarsans. This pattern has been recovered among early tetrapods [64], where dietary innovation rather than terrestriality triggered the evolution of new jaw morphologies. Dietary innovation has also expanded the range of viable morphologies in secondarily aquatic tetrapods; the novel feeding modes of extant cetaceans have promoted the evolution of extreme jaw shapes not seen in terrestrial mammals [65,66]. Crurotarsans, however, did not evolve any such novel diets or feeding modes in the aquatic realm, and so did not expand their occupation of morphospace in the same way that placental mammals have done. Dietary signal in crurotarsans is most strongly linked with the optimizing rotational efficiency, as seen in piscivorous taxa, though durophages also show reasonably high convergence.

## 5. Conclusions

Crurotarsi show clear functional signals induced by environment and diet that have driven their morphological evolution. The classical picture of a functional trade-off between jaw strength and speed is somewhat corroborated by our abstracted models, along with an additional hydrodynamic constraint on aquatic taxa. Comparing the functional properties of our theoretical shapes with realized jaws, we suggest that jaw strength acts mainly as a hard boundary against weaker jaws rather than as a strong driving factor. Specialist feeders such as durophages and herbivores may benefit from increased strength but overlap with other dietary categories suggests that this selection is not overwhelmingly strong. Diet seems to be a greater indicator of jaw shape than environment, especially for fish-eating taxa, though hydrodynamic restrictions prevent marine forms from exploring the entire range of morphospace occupied by terrestrial taxa. Here, we provide a strong collection of evidence that the evolutionary history of crurotarsans is rich with functional innovation and sheds light on the factors that have driven the diversification of this long-lasting clade.

This study highlights the role that function plays in jaw morphological evolution. For stress resistance and hydrodynamic efficiency, function operates as a constraint prohibiting the evolution of suboptimal shapes but allowing the exploration of many equally optimal shapes. Relatively few theoretical shapes are optimized for rotational efficiency and so any strong selection pressure on jaw closure speed creates constraints on jaw morphology, an idea supported by the distribution of empirical taxa across our theoretical performance surface. These functions do not explain all aspects of the spread of realized shapes though, and untested functions, phylogenetic influence, competition from other clades or simple chance likely also influenced the morphology of crurotarsan jaws. Comparison between realized and theoretical jaw shapes, even significantly simplified ones, and the opportunity to test functional hypotheses outside of existing biological structures is a hugely valuable tool for understanding the interaction between form and function.

## Data Availability

All MATLAB script used for this study, alongside all the tif files of jaw outlines in our sample, can be found on Dryad [35]. The underlying code and functions to perform the analyses in MATLAB are available on Github [67]. Supplementary material is available online [68].
